# Caspofungin-Loaded Formulations for Treating Ocular Infections Caused by *Candida* spp.

**DOI:** 10.3390/gels9040348

**Published:** 2023-04-20

**Authors:** Noelia Pérez-González, María J. Rodríguez-Lagunas, Ana C. Calpena-Campmany, Nuria Bozal-de Febrer, Lyda Halbaut-Bellowa, Mireia Mallandrich, Beatriz Clares-Naveros

**Affiliations:** 1 Department of Pharmacy and Pharmaceutical Technology, Faculty of Pharmacy, Campus of Cartuja, University of Granada, 18071 Granada, Spain; noeliaperez93@correo.ugr.es (N.P.-G.); beatrizclares@ugr.es (B.C.-N.); 2 Department of Biochemistry & Physiology, Faculty of Pharmacy & Food Sciences, Universitat de Barcelona (UB), 08028 Barcelona, Spain; 3 Nutrition and Food Safety Research Institute (INSA-UB), 08921 Santa Coloma de Gramenet, Spain; 4 Department of Pharmacy and Pharmaceutical Technology and Physical Chemistry, Faculty of Pharmacy and Food Sciences, Universitat de Barcelona (UB), 08028 Barcelona, Spain; 5 Institute of Nanoscience and Nanotechnology (IN2UB), Universitat de Barcelona (UB), 08028 Barcelona, Spain; 6 Department of Biology, Healthcare and the Environment, Faculty of Pharmacy and Food Sciences, Universitat de Barcelona (UB), 08028 Barcelona, Spain; 7 Biosanitary Institute of Granada (ibs.GRANADA), 18012 Granada, Spain

**Keywords:** caspofungin, ocular drug delivery, antifungal activity, ocular gels, poloxamer 407, ocular infection

## Abstract

Fungal keratitis causes corneal blindness worldwide. The treatment includes antibiotics, with Natamycin being the most commonly used; however, fungal keratitis is difficult to treat, so alternative therapies are needed. In situ gelling formulations are a promising alternative; this type of formulation has the advantages of eye drops combined with the advantages of ointments. This study was designed to develop and characterize three formulations containing 0.5% CSP: CSP-O1, CSP-O2, and CSP-O3. CSP is an antifungal drug that acts against a diverse variety of fungi, and Poloxamer 407 (P407) is a polymer of synthetic origin that is able to produce biocompatible, biodegradable, highly permeable gels and is known to be thermoreversible. Short-term stability showed that formulations are best stored at 4 °C, and rheological analysis showed that the only formulation able to gel in situ was CSP-O3. In vitro release studies indicated that CSP-O1 releases CSP most rapidly, while in vitro permeation studies showed that CSP-O3 permeated the most. The ocular tolerance study showed that none of the formulations caused eye irritation. However, CSP-O1 decreased the cornea’s transparency. Histological results indicate that the formulations are suitable for use, with the exception of CSP-O3, which induced slight structural changes in the scleral structure. All formulations were shown to have antifungal activity. In view of the results obtained, these formulations could be promising candidates for use in the treatment of fungal keratitis.

## 1. Introduction

Ocular infections caused by fungi, bacteria, parasites, or other pathogens are one of the primary causes of blindness in the world, particularly in developing countries [1]. Most corneal ulcers are caused by fungal infections [2,3,4]. It has a high morbidity rate, as people who suffer from it are left with a high level of visual impairment. The most common pathogens are *Candida*, *Aspergillus,* and *Fusarium* spp. Fungal keratitis is very difficult to treat and is characterized by a poor response to antifungal treatment, requiring surgical intervention [5]. The standard treatment for establishing the diagnosis of fungal keratitis is a corneal scraping for microscopic examination and culture preparation. As soon as the diagnosis is confirmed, treatment with an antibiotic that covers the causative microorganism should be started without delay. Natamycin is the most widely used drug. However, it has not been shown to be effective in the treatment of keratomycosis. For this reason, voriconazole is a better alternative [6]. The current recommendation in the literature is to initiate biased antifungal therapy, with the treatment regimen being individualized according to the patient’s response to therapy [7,8].

In the therapeutic arsenal, a limited number of eye drops with antifungal action are available for the treatment of fungal keratitis. This fact, coupled with the poor response to antifungal treatment, creates the need to develop new topical antifungal agents that are currently available intravenously [9].

**Figure 1:**
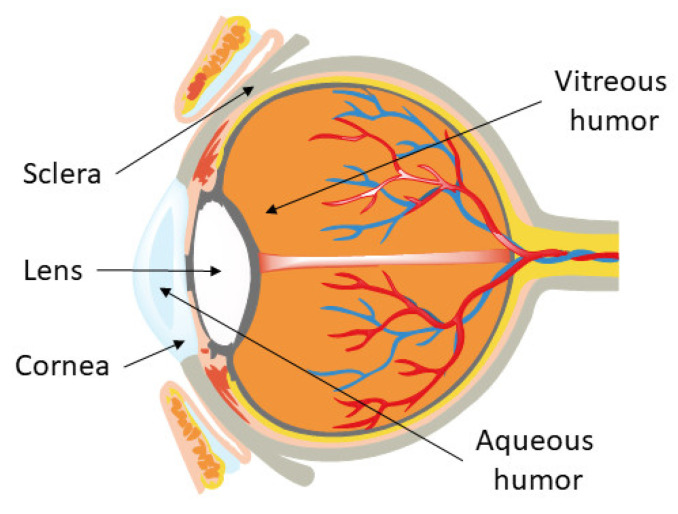
Anatomical structure of the eyeball.

A new family of antifungal drugs is now available: the echinocandins, of which caspofungin (CSP) is the most remarkable [10,11,12]. CSP is a semi-synthetic, water-soluble lipopeptide derived from a fermentation product of *Glarea lozoyensis*. The mechanism of action is to block the synthesis of (1,3)-D-glucan, an essential element of the cell wall of many fungal species [13,14,15,16]. CSP exhibits activity against a diverse range of fungi, with MIC_90_ values in the range from 60 ng/mL to 2000 ng/mL [17,18]. Although clinically, CSP is administered intravenously, CSP eye drops have been reported to be effective for keratitis in rabbits [19,20] and in some patients [21,22,23]. The use of release systems for vaginal administration of CSP has already been reported [24]. The validity of CSP as an agent used to treat fungal keratitis is unknown, as no evidence is available on its ability to permeate into the aqueous humor (Figure 1) when administered topically [25].

The most commonly used method of topical administration of eye drugs is instillation. The eye drops have the disadvantage that they are easily washed out of the area as a result of tearing; they are retained for no more than 2 min, and the bioavailability of the active ingredients is less than 5% [26]. An alternative to increasing the contact time of the formulation is to increase the viscosity of the formulation, but this often leads to blurred vision in the case of ointments or lack of patient compliance. The strategy to resolve this problem is to develop preparations that will gel in situ on contact with the eye and thus achieve a longer residence time [27]. These delivery systems combine the advantages of eye drops—reproducibility of the required dose, simplicity of administration and accuracy—and gels—longer contact time with the affected area [28]. Poloxamer 407 (P407) is a polymer of synthetic origin able to produce biocompatible, biodegradable, and highly permeable gels [29]. P407 is a hydrophilic nonionic surfactant and is formed by polypropylene oxide (PPO) as the core block and poly(ethylene oxide) (PEO) as flankers to form the PEO-PPO-PEO structure known to be thermoreversible in function of the concentration and the temperature employed [30]. The incorporation of CSP into the polymeric matrix of P407 will protect it from degradation, enhancing the therapeutic effect. In addition, P407 is capable of forming micelles that could provide the system with exceptional solubility [31].

In the present investigation, three topical ocular delivery systems of CSP for the treatment of fungal keratitis have been developed and optimized. One eye drop is based on physiological saline solution, and the rest are based on P407 in their composition, with the intention that it gels in situ on contact with the eye and increases bioavailability in the deep ocular tissue. All of them incorporate CSP at 0.5%. Short-term stability, rheological analysis, in vitro release, and permeation profiles were conducted. Ocular tolerance and corneal transparency studies have been carried out with the aim of demonstrating that none of the formulations developed causes eye irritation through tolerability. Histological and antifungal studies were also conducted.

## 2. Results and Discussion

### 2.1. Physicochemical Characterization

Table 1 reports the pH values obtained from the formulations freshly prepared (t_0_) and after three weeks (t_21_) stored at 4, 25, and 32 °C. It could be noted that after three weeks, the pH values remained constant. No significant differences were recorded between the same formulation after storage at the different temperatures.

Freshly prepared samples (t_0_) were transparent, and no precipitates were observed after 21 days. After three weeks, all samples maintained their initial appearance when stored at 4 °C. CSP-O1 turned slightly yellow at 25 and 32 °C. In contrast, both CSP-O2 and CSP-O3 maintained their initial appearance at 25 °C but not at 32 °C, turning slightly yellow.

The fact that samples CSP-O2 and CSP-O3 maintain their appearance at 4 and 25 °C but not at 32 °C may be due to the micelles formed by P407. P407 is known to be a thermoresistant polymer, and the micelles it forms can assemble and dissociate as a function of temperature [32,33,34]. At 32 °C, they may dissociate into monomers and expose the CSP to degradation, resulting in formulations with a slight yellow color. The same may occur with CSP-O1, since this formulation does not contain P407 and therefore leaves the CSP more exposed to possible degradation, hence the yellow color at 25 and 32 °C.

Table 2 displays the rotational test readings of the formulations measured at 4 and 25 °C to simulate cold and room temperature storage conditions, respectively, and 32 °C to simulate eye application conditions.

**Figure 2:**
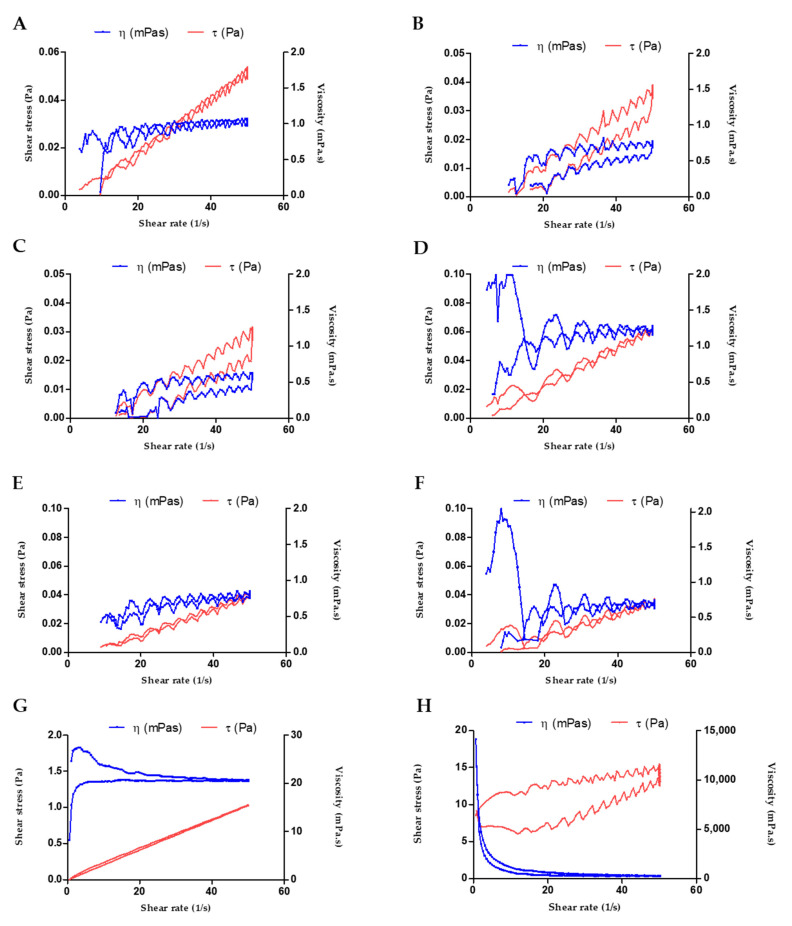

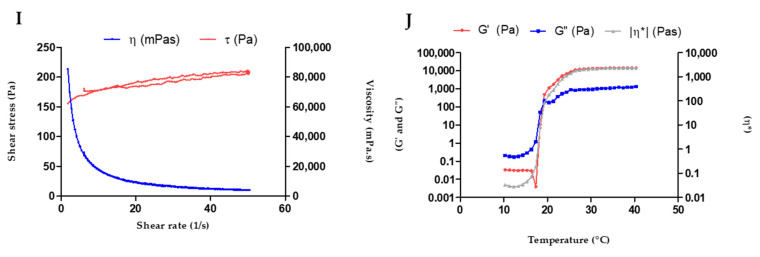
Flow and viscosity curves of samples: (**A**) CSP-O1 at 4 °C; (**B**) CSP-O1 at 25 °C; (**C**) CSP-O1 at 37 °C; (**D**) CSP-O2 at 4 °C; (**E**) CSP-O2 at 25 °C; (**F**) CSP-O2 at 37 °C; (**G**) CSP-O3 at 4 °C; (**H**) CSP-O3 at 25 °C; (**I**) CSP-O3 at 37 °C; and (**J**) SOL-GEL temperature transition of CSP-O3 hydrogel.

The CSP-O1 (Figure 2A) and CSP-O2 (Figure 2B) formulations exhibited Newtonian behavior in all three thermal test conditions, as the flow curve profiles best fitted Newton’s equation. The viscosity of CSP-O2 was slightly higher, due to the presence of 0.2% P407. The increase in temperature implied a decrease in viscosity for both formulations.

In contrast, CSP-O3 (Figure 2C–E) is the clear example of a P407-based formulation: it behaves as a liquid at cold temperatures and as a gel at temperatures that are close to the temperature of the body [35]. The viscosity of the CSP-03 hydrogel increased from 20.50 ± 0.056 mPa·s (SOL structure at 4 °C) to 4166 ± 32.72 mPa·s (GEL structure at 32 °C). In parallel, the flow curve of the CSP-03 hydrogel exhibited Newtonian behavior at 4 °C and a non-Newtonian character at 32 °C, corresponding to a shear thinning (pseudoplastic) profile best described by the Cross equation, according to the change in the system structure.

Moreover, the CSP-O3 flow curve showed apparent thixotropic behavior at 25 and 32 °C as the rheograms displayed a hysteresis loop. These rheological properties are suitable for ocular administration, as this in situ gelling ophthalmic drug delivery system contributes to the formation of a stable and uniform film covering the area of application and allowing the drug to diffuse through the matrix [36,37,38].

The results of the temperature sweep test that was carried out to investigate the in situ gelling of the poloxamer in the case of CSP-O3 gel are visualized in Figure 2F. The T_SOL-GEL_ (where G′ = G″) was about 19 °C. This is the temperature at which the sample exhibits a change from a predominantly viscous behavior (G″ > G′) to a predominantly elastic one (G′ > G″). This interesting gelling process leads to an increased residence time, which avoids the rapid elimination of the gel [39].

**Figure 3:**
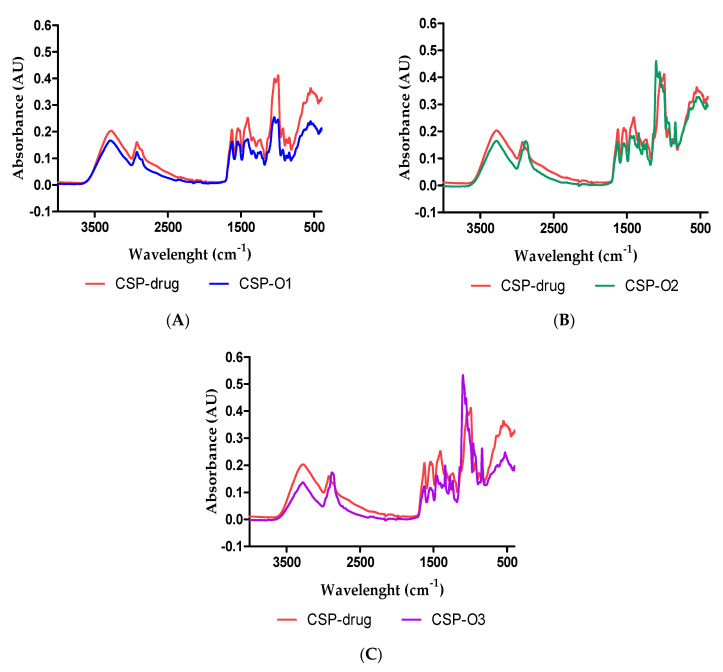
FTIR spectra of: (**A**) CSP drug vs. CSP-O1; (**B**) CSP drug vs. CSP-O2; and (**C**) CSP drug vs. CSP-O3.

The Transform Fourier Infrared (FTIR) was performed to investigate any possible chemical interaction between the drug and the polymers. Figure 3 depicts the FTIR spectra for each formulation related to the CSP drug spectrum. No interaction between the polymer and CPS was observed.

### 2.2. Ocular Hydration (OH) Measurements

**Figure 4:**
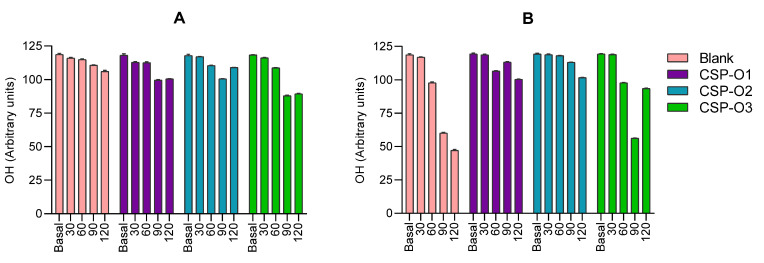
Ocular hydration values obtained after a two-hour application of the samples to (**A**) the corneal membrane and (**B**) the scleral membrane. Each value represents the mean ± SD (*n* = 10). All formulations at all times, with the exception of basal, show statistically significant differences (*p* < 0.001).

This project has described for the first time the parameter that measures the basal hydration values of ocular tissue ex vivo and after applying different formulations. Figure 4 shows the ocular hydration (OH) results obtained after the application of the formulations to the corneal membrane (Figure 4A) and scleral membrane (Figure 4B).

Progressive dehydration was observed over the 2 h of monitoring, especially in the blank sclera. This is probably due to the fact that the measurements were carried out on ex vivo eyeballs, which do not retain moisture through tears and blinking as would in vivo eyes. The general tendency is that hydration is decreased in all cases, being more pronounced when CSP-O3 was applied to both corneal and scleral tissue. In scleral tissue, a marked dehydration of the tissue can be observed, a phenomenon that does not occur when the different formulations are applied. In this case, the hydration appears to have been maintained over time. In general terms, it can be said that CSP-O1 and CSP-O2 act to protect the cornea and sclera from dehydration.

Baseline corneal and scleral values increase with time (2 h). When CSP-O1 and CSP-O3 were applied to the corneal tissue, a marked increase was observed in the first half hour with regard to the blank tissues. The increase is probably due to the water content in the formulations that moisten the tissue surfaces when applied. CSP-O1 showed similar TOWL values to the blank cornea and sclera, then decreased and increased again. The CSP-O2 formulation increased the values in the cornea, with some slight decreases observed between one and one and a half hours. All formulations showed similar behavior when applied to the scleral tissue, with lower TOWL results in CSP-O2.

In general, the values obtained for both tissues show that TOWL increases after being in contact with the formulations for 2 h.

### 2.3. Transocular Water Loss (TOWL) Measurements

**Figure 5:**
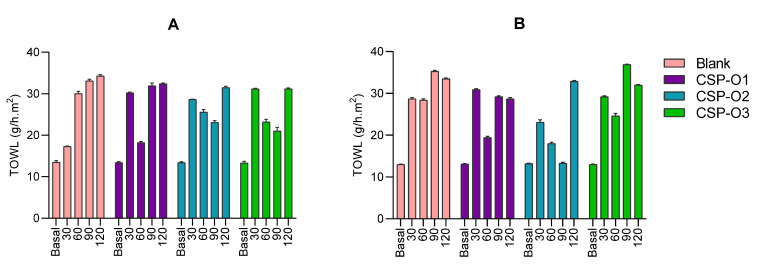
TOWL obtained after a two-hour application of the samples to (**A**) the corneal membrane and (**B**) the scleral membrane. Each value represents the mean ± SD (*n* = 10). All formulations at all times, with the exception of basal, show statistically significant differences (*p* < 0.001).

Transocular water loss (TOWL) is a method based on the measurement of transepidermal water loss (TWEL) to determine the integrity of the barrier function of the skin. Similarly, TOWL could be used as a tool to evaluate the integrity of corneal and scleral tissue after application of different formulations. This is the first time that the parameter measures ex vivo baseline values in porcine corneal and scleral tissue as well as values after application of CSP-loaded formulations. Figure 5 shows the values for TOWL in both corneal (Figure 5A) and scleral (Figure 5B) tissue after a two-hour application of the samples. The ocular dehydration resulted in increased Transocular Water Loss (TOWL), which measures water evaporation. When analyzing the application of the formulations to the cornea, it was observed that the increasing pattern of TOWL over time was modified in the early times post-application, especially the first 30 min, in which the TOWL values were higher than the blank, probably because of the water content in the formulations. Formulation CSP-O1 showed similar TOWL values to the blank cornea from 90 min on, and the same effect of the formulation was observed on the sclera. Regarding formulations CSP-O2 and CSP-O3, the content of pluronic in their composition suggests that the polymer would minimize the transocular water evaporation during the first 60 min regardless of pluronic concentration since both formulations exhibited a similar behavior on the cornea, whereas CSP-O3 presented a greater capacity in preventing the transocular water loss in the sclera than CSP-O2. This might be due to the structural differences between corneal and scleral tissue [40].

### 2.4. In Vitro Release and Kinetic Evaluation

**Figure 6:**
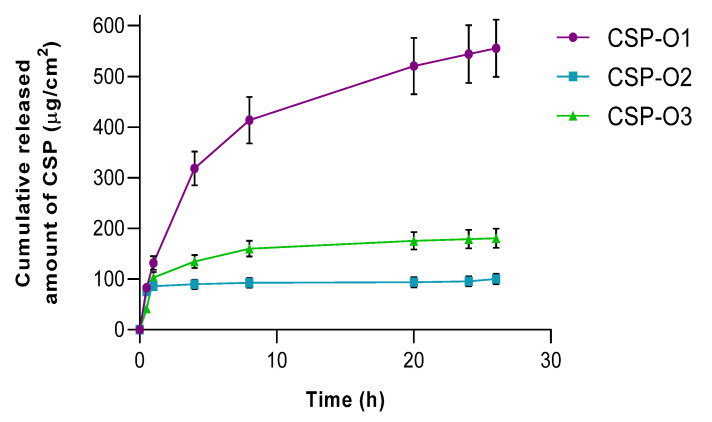
Cumulative amount released of CSP from formulations plotted against time. Data represents mean ± SD (*n* = 5).

The release profiles of formulations are shown in Figure 6. The fitting results revealed that the samples conformed to the one-site binding model, Equation (1):

(1) $$Y=\frac{B_{max}\cdot X}{K_{d}+X}$$

where *B_max_* is the maximum amount that can be released and *K_d_*, is the time needed to reach 50% of the drug released.

In view of the results reported, it can be said that CSP-O1 releases CSP most rapidly, CSP-O3 is the second fastest releaser, and finally CSP-O2. During the first hour, all formulations show a fast release. This is followed by a slower release until the end of the test. Statistical analysis showed significant differences for the *K_d_* value, which means that each formulation releases 50% of the drug in a different way.

Table 3 shows the values obtained for the model parameters, in addition to the values for the non-modelistic parameters such as the mean dissolution time (MDT, the mean dissolution time of CSP in the formulation throughout the process), the area under the curve (AUC, representing the amount of CSP released from the formulation), and the efficiency (E). CSP-O2 presented the highest MDT value, 21.7 ± 2.4 h, followed by the CSP-O3 formulation with a value of 18.7 ± 1.7 h, and at last CSP-O1 with a value of 6.1 ± 0.5 h. Based on these results, CSP-O2 would be the formulation that releases CSP the fastest, suggesting that it is the formulation that can provide the best bioavailability of the drug in ocular tissue. In terms of AUC values, the highest value corresponds to CSP-O1, 11,040.0 ± 1087.0 µg/cm^2^·h, while the lowest value corresponds to CSP-O2, 2381.0 ± 248.0 µg/cm^2^·h. Finally, the CSP-O1 formulation showed an E value of 76.5 ± 7.1%, the CSP-O2 a value of 16.5 ± 1.3%, and 28.2 ± 2.6% for CSP-O3.

All formulations are able to release the drug, so permeation of the drug through the corneal and scleral membranes should not be limited.

### 2.5. Ex Vivo Permeation of CSP through Ocular Mucosa

**Figure 7:**
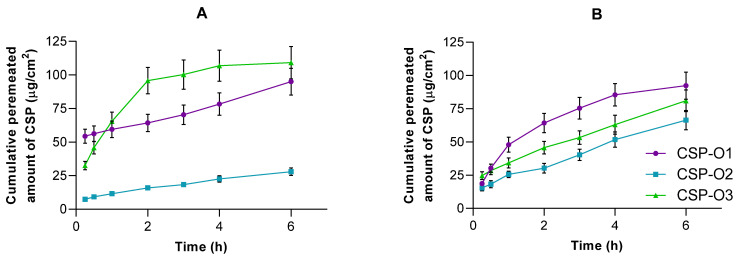
Cumulative amount of CSP permeated (µg) through (**A**) the corneal membrane and (**B**) the scleral membrane upon application of CSP formulations. Each value represents the mean ± SD (*n* = 6).

Figure 7 shows the permeation profiles of all formulations when they are passed through the corneal membrane (Figure 7A) and scleral membrane (Figure 7B). The CSP permeation trend was different in both tissues. The CSP permeation tendency was different in both tissues. In the cornea, the formulations fit a one-site binding release profile. CSP-O3 permeated the most, followed by CSP-O1, and finally CSP-O2. The permeation parameters were calculated in the linear section, i.e., in the first hour, and can be seen in Table 4.

On the other hand, in the sclera tissue, CSP-O1 permeated the fastest, followed by CSP-O3, and again, CSP-O2 permeated the least. In this case, both CSP-O2 and CSP-O3 were fitted to a linear curve, while CSP-O1 was fitted to a single-site binding model. The permeation parameters were calculated in the linear section of CSP-O1, even though CSP-O2 and CSP-O3 are already linear, as can be seen in Table 4.

The fact that all formulations are able to permeate through tissue indicates that CSP will pass into the aqueous humor through the cornea and into the vitreous humor through the sclera.

The amount of CSP retained (*Q_r_*) in the cornea and sclera was also calculated (Table 4). For CSP-O1, the amount retained in the cornea was 28.83 ± 2.81 µg/cm^2^ and 48.79 ± 5.23 µg/cm^2^ for the scleral tissue. For the CSP-O2 formulation, we found 11.47 ± 1.79 µg/cm^2^ and 30.61 ± 3.47 µg/cm^2^, for corneal and scleral tissue, respectively. Finally, for CSP-O3, the amounts retained for the cornea and sclera were 7.03 ± 0.75 µg/cm^2^ and 20.09 ± 2.43 µg/cm^2^. In view of the results obtained and probably due to the difference in thickness between the two tissues, more CSP is retained in the sclera than in the cornea. [41,42].

It should be noted that this method is static and would not resemble the real conditions of application of the formulation, so in vivo studies would be necessary to help objectively discard invalid formulations. To date, no previous studies have been reported where the permeation parameters of CSP through ocular tissue have been calculated. CSP-O1 would be the formulation capable of retaining significant amounts of CSP in both corneal and scleral tissue, suggesting that it is the best formulation to ensure local action.

### 2.6. Histological Evaluation

**Figure 8:**
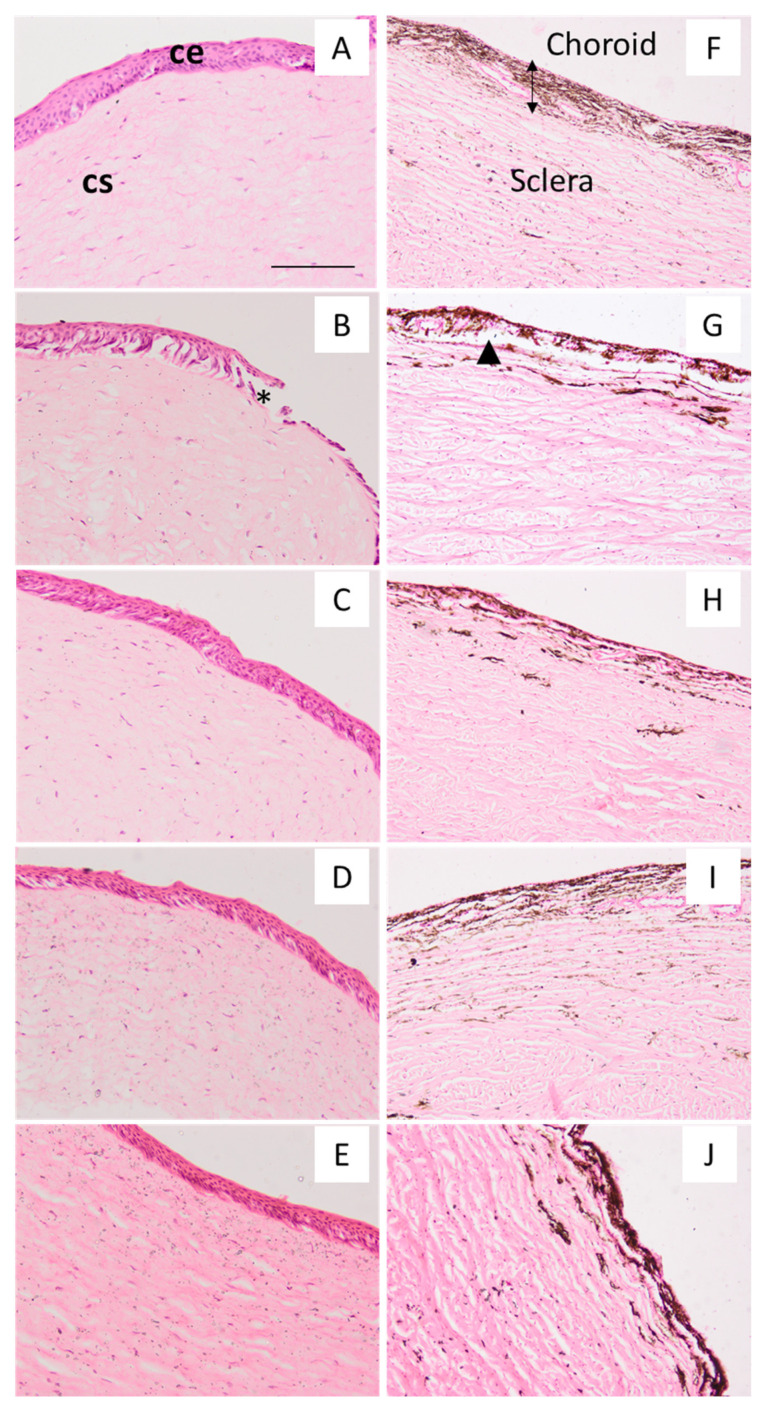
Cornea and sclera sections observed under the microscope. For histological assessment, sections of the cornea (**A**–**E**) and sclera (**F**–**J**) were stained with hematoxylin and eosin and photographed at 200×and 100×, respectively. After the ex vivo permeation assay, tissues were treated with physiological serum (**A**,**F**), ethanol-water 50:50 (**B**,**G**), CSP-O1 (**C**,**H**), CSP-O2 (**D**,**I**), and CSP-O3 (**E**,**J**). ce—corneal epithelium; cs—corneal stroma (lamina propria). Asterisk indicates a loss of the corneal epithelium, and the arrowhead indicates a disruption of the choroid. Scale bar: 200 µm (100×) and 100 µm (200×).

For histological analysis, the cornea and sclera were treated with serum as a control condition (Figure 8A,F) and with ethanol and water (50:50) as a positive control (Figure 8B,G). The ethanol dilution induced the loss of corneal epithelium and disrupted the choroid, which is the upper layer of the sclera and rich in blood vessels, melanocytes, and fibrocytes. The treatment with CSP-O1 (Figure 8C,H), CSP-O2 (Figure 8D,I), and CSP-O3 (Figure 8E,J) formulations did not induce remarkable changes in the corneal epithelium; only CSP-O3 induced the loosening of the corneal stroma, which was also present in positive control conditions. With regard to the sclera, only CSP-O3 induced the contraction of the choroid and the loosening of the sclera. Overall, the CSP-O2 formulation was the one that showed the fewest histological changes, followed by the CSP-O1 formulation.

Although these results indicate that the formulations, with the exception of CSP-O3, are suitable for use and did not induce significant changes in the corneal epithelium, in vivo studies are still needed to confirm if similar results are obtained.

### 2.7. Antifungal Efficacy

**Figure 9:**
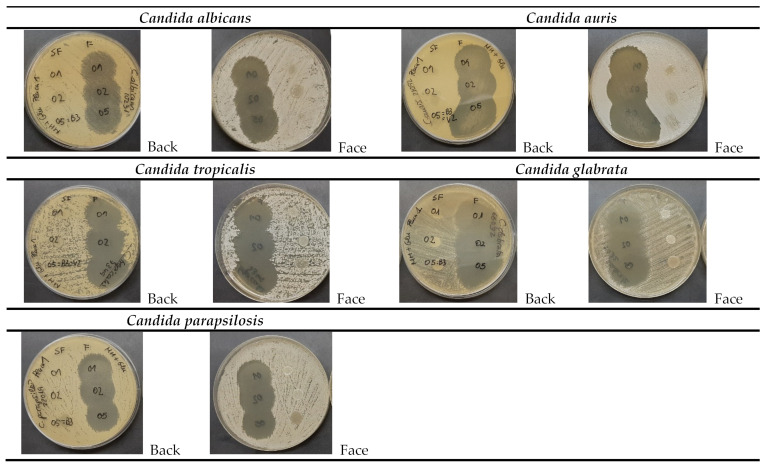
Inhibition zones produced by formulations with and without 0.5% caspofungin against yeast *C. albicans* ATCC 10231, *C. auris* DSM 21092, *C. tropicalis* ATCC 7349, *C. glabrata* ATCC 66032, and *C. parapsilosis* ATCC 22019. In a frontal view (face), the Caspofungin formulations marked “F” are seen on the left of the plate, and the excipients O1, O2, and O3 marked “SF” on the right, whereas in a view from the reverse (back) of the plate, positions are reversed.

Caspofungin is known to be effective against several *Candida* strains [43]. In this work, CSP was loaded into three different formulations (CSP-O1, CSP-O2, and CSP-O3), and a qualitative antifungal efficacy test was conducted to verify that the formulations maintained the antifungal effect. The study has shown that the CSP-loaded formulations are effective against most of the tested *Candida* spp., and the antifungal action of CSP was highly effective against four out of five *Candida* strains tested, with the exception of *C. auris* strain DSM 21092, where we found that the formulations strongly inhibited the yeast growth for the first 24 h of incubation but a small resistant growth appeared at 48 h (Figure 9). In general, all formulations were able to produce large areas of inhibition, evidencing the efficacy of these formulations.

As for the formulations without CSP load (O1, O2, and O3), it was observed that the high-concentration poloxamer-based formulation (O3) prevented the growth of the *Candida* strains tested on the application zone (Table 5). Our results are in line with the previous reported efficacy of these types of formulations against fungal infections. It is known that there are synergistic effects between P407 and the antifungal agents [44]. Table 5 summarizes the results obtained.

### 2.8. In Vitro Tolerance Study

**Figure 10:**
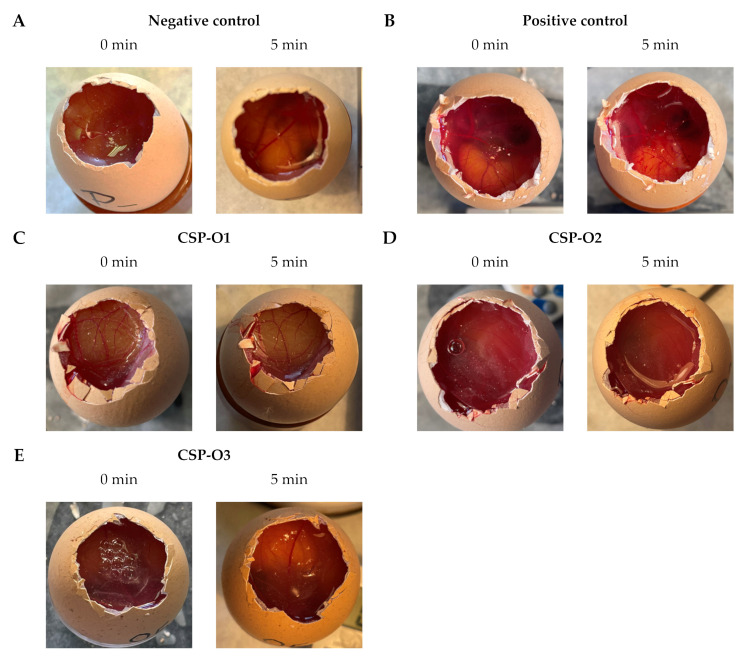
Results of the HET-CAM test: (**A**) physiological saline solution as the negative control; (**B**) NaOH 0.1 N as the positive control; (**C**) CSP-O1 formulation; (**D**) CSP-O2 formulation; and (**E**) CSP-O3 formulation.

After application of formulations to the chorioallantoic membrane of 10-day-old embryonic eggs, no irritating effect was observed after 5 min. The results are shown in Figure 10. Physiological saline solution, which is known to be non-irritant, and 0.1 N sodium hydroxide (NaOH) solution, which produced hemorrhage, coagulation, and coagulation by lysis of blood vessels from the onset, were used as controls.

After the HET-CAM test, the irritation score (IS) of CSP-loaded formulations was calculated. The results obtained are reported in Table 6. All formulations showed values below 0.09, therefore all are classified as non-irritating.

**Figure 11:**
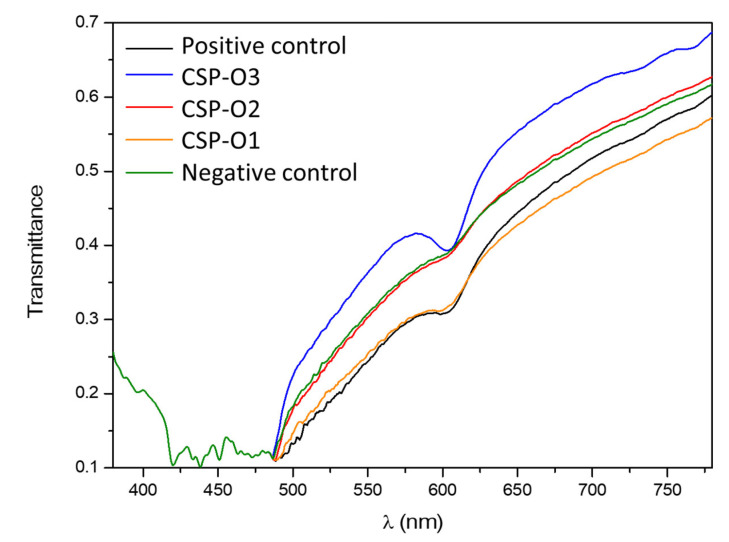
Transmittance profile from 400 to 800 nm wavelength of corneas treated for 10 min with ethanol as positive control, physiological saline solution as negative control, CSP-O1; CSP-O2; and CSP-O3.

To contrast the information obtained by HET-CAM, the transparency of the corneas was observed when the formulations were applied. The transmittance profile can be seen in Figure 11. A physiological saline solution was used as a negative control, and ethanol, which reduced corneal transparency, was used as a positive control. All formulations have the same spectrum at 485 nm and then change. A relative minimum is also observed in all of them between 600 and 605 nm. CSP-O1 does not significantly differ from the positive control, CSP-O2 is very similar to the negative control, and CSP-O3 lets more light through than the control. The transmittance profiles of CSP-O2 and CSP-O3 indicate that both formulations do not affect corneal transparency.

The chorioallantoic membrane is very sensitive to chemicals [46,47]. The HET-CAM test is suitable to test for potential eye irritation after application of the formulations. All formulations have proven to be non-irritating and should therefore be well tolerated. However, changes in corneal transparency were observed with CSP-O1. CSP-O3 proved to be better than CSP-O2. Additional studies are still needed to evaluate formulations without the need for human testing and to exclude formulations more objectively. One of the techniques that could be used is synchrotron X-ray scattering analysis to assess the structure of collagen in the cornea, which is responsible for transparency [48,49]. It should be noted that most methods are static and would not correspond to the real conditions of application of the formulation, so in vivo studies would be necessary to help objectively contrast the results obtained.

## 3. Conclusions

Three formulations containing CSP were designed and characterized: CSP-O1, CSP-O2, and CSP-O3. Physiological saline was used to prepare CSP-O1, while the other two were made with P407 at 0.2% and 18%, respectively. All of them with CSP at 0.5%.

Short-term stability showed that the formulations should be kept at 4 °C. However, CSP-O2 and CSP-O3 showed to be also stable at 25 °C. Rheological studies revealed that CSP-O3 was able to gel in situ while CSP-O2 was not due to the low concentration of P407 in its composition. TOWL values increased in all cases, whether on blank eyes or treated eyes, when applying the formulations to the eyeball. A remarkable increase in the first 30 min was observed with respect to the blank, probably because of the water content in the formulations. Progressive dehydration of the cornea, especially the scleral tissue, was observed over time. Formulations CSP-O1 and CSP-O2 minimized this effect.

The CSP release study followed the one-site binding model, showing that CSP-O1 is the formulation that releases the most CSP as well as the formulation that retains the most CSP in the ocular tissue. However, permeation studies showed that CSP-O3 is the formulation that permeates the most through the cornea and CSP-O1 through the scleral tissue. Histological sections indicated that CSP-O3 induced corneal stromal loss, while the others did not induce significant changes in the corneal epithelium. The antifungal activity showed that all formulations inhibited the growth of *C. albicans*, *C. glabrata*, *C. parapsilosis*, and *C. tropicalis*, but *C. auris* showed to be resistant to the CSP formulations. Finally, the formulations proved to be non-irritating to the chorioallantoic membrane, and CSP-O2 and CSP-O3 did not alter corneal transparency, an important factor to take into account to ensure patient adherence to treatment. All these findings suggest that, among all the three formulations investigated, CSP-O2 appears to be the most promising for topical ocular CSP delivery.

## 4. Materials and Methods

### 4.1. Materials

#### 4.1.1. Chemical and Reagents

CSP acetate salt was supplied by SunPharma (Barcelona, Spain). Trifluoroacetic acid and methanol were acquired from Sigma Aldrich (Madrid, Spain). Pluronic^®^ F-127 (P407) was provided by BASF (Barcelona, Spain). Double-distilled water was obtained from a Milli-Q^®^ Gradient A10 system apparatus (Millipore Iberica S.A.U.; Madrid, Spain). Physiological saline solution was purchased by Grifols Laboratories S.A. (Barcelona, Spain). Ketamine HCl was supplied by Pfizer (Madrid, Spain). Xylazine was acquired by Laboratories Calier (Barcelona, Spain). Midazolam, propofol, and sodium thiopental were supplied by B. Braun Medicals (Barcelona, Spain). Isoflurane was purchased from Baxter (Valencia, Spain). Polytetrafluoroethylene (PTFE) membranes were provided by Pall^®^ Corporation (Madrid, Spain). All other chemicals and reagents used in this study were HPLC-grade, unless otherwise stated.

#### 4.1.2. Biological Tissue for Ex Vivo Assays

Corneal and scleral membranes were acquired from female pigs weighing approximately 25 kg. Animals were sacrificed under veterinary supervision at the Animal Facility of the Bellvitge Campus of the University of Barcelona (Spain) and with the agreement of the Animal Experimentation Ethics Committee of the University of Barcelona, Spain (CEEA-UB) code 10617. Pigs were anesthetized with ketamine HCl (3 mg/kg), xylazine (2.5 mg/kg), and midazolam (0.17 mg/kg). After sedation, propofol (3 mg/kg) was administered, and then the patient was intubated and maintained under inhaled anesthesia with isoflurane. Finally, with an overdose of thiopental, the animals were euthanized.

The cornea and sclera of the animals were cut away, placed in an artificial tear solution, and refrigerated until experiments. Before use, ocular tissues were superficially cleaned with gauze soaked in a 0.05% dodecyl sulfate solution, followed by distilled water.

### 4.2. Preparation of CSP-Loaded Formulation

In this study, three formulations of loaded CSP were designed and developed. A physiological saline solution was chosen to develop the first formulation (CSP-O1). CSP-O1 was obtained by solubilizing of 0.5% (*w*/*v*) CSP in physiological saline solution.

It was decided to use P407 due to its amphiphilic nature for the remaining formulations. P407 is a copolymer consisting of a hydrophobic polypropylene oxide (PPO) core block flanked by hydrophilic polyethylene (PEO) blocks to give a final PEO-PPO-PEO structure. It is this structure that gives it the ability to solubilize active ingredients through the formation of micelles. As CSP is a water-soluble drug, it would be located in the PEG block [34,50].

CSP-O2 was prepared as follows: a concentration of 0.2 % (*w*/*v*) P407 and 0.5 % (*w*/*v*) CSP was mixed and stirred at 4 °C until it was homogenized.

A concentration of 18% (*w*/*v*) P407 was initially dissolved and stirred overnight at 4 °C (O3). In order to obtain a CSP-loaded P407 hydrogel (CSP-O3), 0.5% (*w*/*v*) CSP was added to O3. The formation of this hydrogel is explained in previous studies of the group [24].

No insoluble particles were visually detected after sample preparation. Thus, all formulations were stored at 4 °C.

### 4.3. Physicochemical Characterization

#### 4.3.1. pH Measurements and Appearance

A digital pH/mV meter, pH 200 (Crison Instruments S.A., Barcelona, Spain), was used for pH evaluation. Measurements of the formulations were made at 25 °C, with the formulations freshly prepared and stored at 4, 25, and 32 °C for three weeks after preparation. Values were recorded as the mean ± SD of three replicates.

Samples stored at 4, 25, and 32 °C were also visually inspected for physical appearance. Parameters such as color or tendency to spontaneously precipitate could be appreciated.

#### 4.3.2. Rheological Behavior

A Haake RheoStress 1 rheometer connected to a Thermo Haake Phoenix II + Haake C25P temperature control (Thermo Fisher Scientific, Karlsruhe, Germany) was employed for rheological characterization of samples. It was equipped with a parallel plate geometry configuration, including a fixed bottom plate and a movable Haake PP60Ti top plate (60 mm diameter), and operated with Haake RheoWin^®^ Job Manager and Data Manager v. 4.87 software.

Each sample was equilibrated for approximately 5 min until it reached the operating temperature by placing it between the plate-plate sensor system, 0.5 mm apart, to determine viscosity and flow behavior. They were subjected to a three-stage shear profile test after reaching the desired temperature of 4, 25, and 32 °C: an increase period (0–50 s^−1^) for 3 min, a period of constant shear rate at 50 s^−1^ for 1 min, and a decrease period (50–0 s^−1^) for 3 min. All determinations were measured in triplicate. Various mathematical models were fitted to the data from the flow curves: Cross, Herschel-Bulkley, Newton, Bingham, Casson, and Ostwald-de-Waele [51]. The model that best described the experimental data obtained was determined based on the best value of the correlation coefficient (r).

Apparent thixotropy was determined by the presence or absence of a hysteresis loop, and viscosity averages (Pa·s) were determined at 50 s^−1^ from the constant share rate period of each viscosity curve. Values are reported as the mean ± SD of three replicates for each formulation and each temperature.

Moreover, the thermosensitive gelation process of the CSP-O3 formulation was researched by ranging the temperature from 10 to 40°C in oscillation mode with a fixed frequency of 1 Hz and a constant voltage of 0.5 Pa. The samples were equilibrated in the same way as for the rotational mode until the initial temperature of 4°C was reached. A controlled ramp rate was used to increase the temperature from 10 to 40°C for 2000 s, and the moduli G′ and G″ as well as the complex viscosity (η*) were recorded. The temperature at which the crossover was detected where G′ = G′′ was established as the gelling temperature or SOL-GEL temperature transition (TSOL-GEL) [27,52].

#### 4.3.3. Fourier Transform Infrared Spectroscopy (FTIR)

A JASCO 6200 (JASCO Analítica Spain, Madrid, Spain) with an attenuated total reflection (ATR) diamond and SPECTRA MANAGER v2 as software were used for FTIR spectra of CSP and formulations. The scanning range was 4000–400 cm^−1^, with a resolution of up to 0.25 cm^−1^.

### 4.4. Ocular Hydration Measurements

The measure of hydration is based on the capacitance of a dielectric medium, utilizing the relatively high dielectric constant of water compared to that of other materials. The corneometer contains two electrodes with different electrical charges that create an electromagnetic field that determines the dielectricity of the stratum corneum.

A Corneometer^®^ 825, mounted on a Multi Probe Adapter^®^ MPA5 (Courage & Khazaka Electronics GmbH, Cologne, Germany), was employed for the measurements. There are no known studies that have determined hydration values in the corneal and scleral membranes, so this would be the first time the term ocular hydration has been described. There are no known studies that have determined hydration values in the corneal and scleral membranes, so this would be the first time the term ocular hydration (OH) has been described. OH values (arbitrary units, AU) were expressed as the mean ± SD of 10 replicates before and after application of the formulations to the cornea and sclera membranes for at least 2 h.

### 4.5. Transocular Water Loss Measurements

Transepidermal water loss (TEWL) is a measure of the amount of water that evaporates and diffuses out of the skin through the epithelium into the surrounding atmosphere. Is a well-established method for the assessment of the integrity of the skin barrier function. Similarly, the integrity of the biological function of the corneal and scleral membranes will be determined using TOWL as an indicator. To date, there are no known studies that have determined TOWL values, so this would be the first time they have been described.

A Tewameter^®^ TM 300 (Courage & Khazaka Electronics GmbH, Cologne, Germany) was used for the measurements. The electrode was positioned on the surface of the tissues for 1 min without any pressure. It is important to maintain optimal room environmental conditions. Therefore, measurements were taken with a relative humidity of 60% and a temperature of 25 °C. TOWL values (g/m^2^·h) were expressed as the mean ± SD of 10 replicates before and after application of the formulations for at least 2 h on the cornea and sclera membrane.

### 4.6. In Vitro Release and Kinetic Evaluation

Vertical diffusion Franz cells (FDC 400, Crown Glass, Somerville, NY, USA) made of amber glass with a receiver chamber volume of 12 mL and an effective diffusion area of 2.54 cm^2^ were used for the CSP release studies. The membrane used was polytetrafluoroethylene (wwPTFE, 0.45 µm; 47 mm). To recreate in vivo ocular conditions, the system was maintained at 32 ± 0.50 °C throughout the test. Physiological saline was used as the receiving fluid, and the whole system was covered with parafilm to prevent evaporation and kept under continuous agitation at 600 rpm.

During different time intervals during the next 28 h, sample aliquots were extracted and analyzed to determine the amount of CSP released. The CSP concentration is presented as the mean ± SD of five replicates. The values obtained were fitted to different kinetic models. The best-fitting model was selected based on the best r^2^ value. Finally, some amodelistic parameters such as MDT, AUC, and E were also calculated.

### 4.7. Ex Vivo Permeation of CSP through Ocular Mucosa

Vertical diffusion Franz cells (FDC 400, Crown Glass, Somerville, NY, USA) made of amber glass with a receiver chamber volume of 5 mL and an effective diffusion area of 0.64 cm^2^ were used for the CSP permeation studies with porcine corneas and sclera membranes as membranes. A known amount of sample was placed in the donor compartment, while the receiver compartment was filled with physiological saline solution. The system was maintained at 32 ± 0.50 °C for the corneal tissue and 37 ± 0.50 °C for the scleral tissue. As in the release study, everything was isolated with parafilm to avoid possible evaporation and left in continuous agitation until the end of the study.

During 6 h at different time intervals, sample aliquots were taken and equal volumes replaced. The samples were analyzed to determine the permeated drug concentration. The amount of CSP permeated through the tissues is expressed as the mean ± SD of six replicates of each tissue for each sample.

The amount of CSP permeated through porcine corneas and sclera per unit area (µg/cm^2^) at each time point was calculated from the concentration of CSP in the receptor fluid and plotted as a function of time (h). The flux (*J*, μg/h) or slope was calculated using Prism^®^ software, V. 5.00 (GraphPad Software Inc., San Diego, CA, USA). The permeability coefficients (*K_p_*, cm/h) of the formulations were calculated using the following equation [53]:

(2) $$K_{p}=J/C_{0}$$

where *C*_0_ is the initial drug concentration in the donor compartment, 5000 μg/mL.

Finally, the cumulative permeated amount of CSP at the end of the study (*A*_6_) was calculated, i.e., the amount accumulated at 6 h. For corneal permeation, *A*_6_ refers to the amount of CSP that would penetrate into the aqueous humor, while for scleral permeation it refers to the amount that would pass into the vitreous humor.

At the end of the permeation study, the tissues were removed and the amount of CSP (*Q_r_*, µg/cm^2^) retained in them was calculated. After removal of the tissues, they were wiped with gauze soaked in 0.05% sodium laurel sulfate and washed with distilled water. This was conducted to remove possible residues from the formulations.

The permeation areas were cut, weighed, and punctured with a fine needle. They were then placed in an ultrasonic bath and immersed in 2 mL of physiological saline solution for 15 min. The samples were analyzed, and the amount of CSP retained (*Q_r_*, µg/cm^2^) is expressed as the mean ± SD of six replicates of each tissue for each sample.

### 4.8. Analysis of CSP in Solution

The samples produced from in vitro release and ex vivo permeation tests were analyzed by a validated UPLC method. The UPLC system consisted of an Acquity I CLASS UPLC System (Waters, Milford, CT, USA) coupled to an Acquity TUV and an Acquity Fluorescence Detector. The excitation and emission wavelengths for fluorescence detection were 224 and 304 nm, respectively. A Lichrospher RP-8 column (125 × 4 mm, 5 µm, Phenomenex) was employed for the separation.

The method was previously validated according to the ICH Q2 (R1) guidelines. Conditions maintained the column at room temperature. The flow rate was 0.8 mL/min for 8 min, and the injection volume was 10 µL. Mobile phase A comprised 0.1% trifluoroacetic acid (TFA) in Milli-Q^®^ water, and mobile phase B consisted of methanol. The gradient elution program was 50–50% B for 0–5 min; 100% B for 5–6 min; and 50–50% B for 6–8 min.

### 4.9. Histological Evaluation

The hematoxylin-eosin stain was used to assess the architecture of the cornea and the sclera treated with CSP-O1, CSP-O2, and CSP-O3 formulations after the ex vivo permeation assay. Following the incubation period, the tissue samples were rinsed in PBS and dehydrated in an increased gradient of ethanol before being placed in xylene and finally embedded in melted paraffin wax. Then tissue blocks were sectioned at 5 µm with a microtome (Leica RM2135), rehydrated, stained with hematoxylin and eosin, and finally mounted with DPX (Sigma). Sections were observed at 100× and 200× magnification under a light microscope (Olympus BX41), and a camera (Olympus XC50) was used for the acquisition of the images. From the six replicates obtained after the ex vivo permeation study, the best corneal and scleral image was selected.

### 4.10. Antifungal Efficacy

The loaded and unloaded formulations of caspofungin were tested to qualitatively examine whether they maintained the antifungal effect. To this end, a modified Kirby-Bauer disc diffusion test [54] was selected, and a similar methodology described in the test protocol [55,56] was followed. The disc diffusion test is based on the diffusion capacity of the antibiotics/antifungals around the area where the sample has been applied.

*C. albicans* ATCC 10231 (American Type Culture Collection, Manassas, VA, USA), *C. glabrata* ATCC 66032, *C. parapsilosis* ATCC 22019, *C. tropicalis* ATCC 7349, and *C. auris* DSM 21092 (German collection of microorganisms and cell cultures GmbH) were used as *Candida* strains for the test. First, they were grown aerobically in Mueller–Hinton (MH) medium supplemented with glucose 2% at 30 °C for 48 h. Inoculum was prepared by suspending colonies in Ringer’s solution to the desired density (0.5 McFarland). The 2% MH-glucose plates were inoculated three times over the entire agar surface with the aid of a yeast-soaked swab, rotating the plate by about 60° each time to ensure even distribution of the inoculum, which could result in a confluent lawn of growth.

Both 0.5% CSP-loaded formulations (CSP-O1, CSP-O2, and CSP-O3) and non-CSP-loaded formulations (O1, O2, and O3) were studied. Nystatin 100 IU/mL and amphotericin B 250 µg/mL were used as controls. Due to the nature of the samples, discs could not be soaked with the samples, and 5 µL of each formulation were deposited on 2% MH-glucose agar inoculated with yeast, and the plates were incubated at 30 °C for 48 h. Each formulation was tested in triplicate for each strain.

### 4.11. In Vitro Tolerance Study

#### 4.11.1. Ocular Irritation Assay: HET-CAM

An in vitro ocular irritation assay was performed to determine the potential irritating properties of the developed formulations. A 10-day-old embryonated hen egg from the G.A.L.L.S.A. farm in Tarragona was used for the HET-CAM test, which measures the ability to induce irritant effects on the chorioallantoic membrane (CAM). The eggs were incubated at a temperature of 37 ± 0.5 °C and a relative humidity of 50–60%. In the experimental procedure, 300 µL of the formulations were applied to CAM, and the degree of severity of each reaction was observed. NaOH 0.1 N was used as the positive control and physiological saline solution as the negative control. The INVITTOX 15 protocol was used for this assay [57]. Both formulations and controls were tested in triplicate.

During the test, the direct observation of hemorrhage (bleeding), coagulation (blood vessel disintegration), and vessel lysis coagulation (protein denaturation intra- and extra-vascular) was performed for 5 min. Each element was considered individually and then combined to obtain a score (*IS*), which was used to classify the irritancy level of the formulation and was calculated using the following equation:

(3) $$IS=\frac{5\cdot \left(301-\sec H\right)}{300}+\frac{7\cdot \left(301-\sec V\right)}{300}+\frac{9\cdot \left(301-\sec C\right)}{300}$$

where sec is the time in seconds when signs started, *H* is the hemorrhage, *V* is vessel lysis, and *C* is the coagulation. The formulations were classified according to the following: *IS* ≤ 0.9, not irritating; 0.9 < *IS* ≤ 4.9, weakly irritating; 4.9 < *IS* ≤ 8.9, moderately irritating; 8.9 < *IS* ≤ 21 irritating [45,58].

#### 4.11.2. Cornea Transparency

To assess changes in corneal transparency after application of the formulations, corneas were exposed to a defined light beam, and transmitted light was detected without absorption or transmission. The transmittance of the corneas was evaluated from 150 to 760 nm after contact with the formulations, physiological saline solution as a negative control, and ethanol as a positive control, for 10 min [41]. Both formulations and controls were tested in triplicate.

### 4.12. Statistical Analysis

Experimental results were statistically examined by one-way analysis of variance (ANOVA) using GraphPad Prism^®^ v. 5.00 software (GraphPad Software Inc., San Diego, CA, USA). The significance level was set at 0.05, and differences between independent groups were determined by applying Tukey’s test and adopting a 95% confidence level.

## Figures and Tables

**Table 1:** pH values in formulations freshly prepared (t_0_) and after three weeks (t_21_), stored at 4, 25, and 37 °C, and their visual appearance. Values represent the mean ± SD (*n* = 3).

Formulation	Temperature (°C)	pH Values over Time					Appearance at t_21_
		pH t_0_	pH t_3_	pH t_7_	pH t_14_	pH t_21_	
CSP-O1	4	6.60 ± 0.02	6.63 ± 0.03	6.59 ± 0.03	6.74 ± 0.02	6.76 ± 0.03	Passed
	25	6.81 ± 0.02	6.77 ± 0.03	6.71 ± 0.02	6.88 ± 0.02	6.75 ± 0.02	Failed
	32	6.56 ± 0.01	6.54 ± 0.01	6.44 ± 0.01	6.53 ± 0.01	6.44 ± 0.01	Failed
CSP-O2	4	6.73 ± 0.07	6.67 ± 0.02	6.84 ± 0.02	6.88 ± 0.03	6.84 ± 0.01	Passed
	25	6.86 ± 0.02	6.93 ± 0.04	6.78 ± 0.04	6.95 ± 0.04	6.80 ± 0.03	Passed
	32	6.62 ± 0.01	6.50 ± 0.01	6.36 ± 0.01	6.55 ± 0.02	6.31 ± 0.01	Failed
CSP-O3	4	6.56 ± 0.01	6.71 ± 0.01	6.70 ± 0.03	6.69 ± 0.09	6.55 ± 0.01	Passed
	25	6.72 ± 0.05	6.78 ± 0.01	6.75 ± 0.01	6.66 ± 0.01	6.43 ± 0.06	Passed
	32	6.72 ± 0.02	6.94 ± 0.01	6.86 ± 0.02	6.65 ± 0.04	6.48 ± 0.01	Failed

**Table 2:** Results of the rheological rotational testing of the formulations. Each value of viscosity represents the mean ± SD (*n* = 3).

Sample		CSP-O1			CSP-O2			CSP-O3		
Temperature (°C)		4	25	32	4	25	32	4	25	32
Rheological behaviour and model	Ramp Up	Newton r = 0.9940	Newton r = 0.9824	Newton r = 0.9839	Newton r = 0.9907	Newton r = 0.9904	Newton r = 0.9816	Newton r = 0.9981	Cross r = 0.9739	Cross r = 0.9390
	Ramp Down	Newton r = 0.9944	Newton r = 0.9884	Newton r = 0.9841	Newton r = 0.9973	Newton r = 0.9934	Newton r = 0.9869	Newton r = 1.0000	Cross r = 0.9712	Cross r = 0.9968
Viscosity at 50 s^−1^ (mPa·s)		1032 ± 0.028	0.738 ± 0.026	0.592 ± 0.026	1.229 ± 0.032	0.806 ± 0.026	0.691 ± 0.031	20.50 ± 0.058	281.7 ± 13.87	4166 ± 32.72
Apparent Thixotropy		Absence	Absence	Absence	Absence	Absence	Absence	Absence	Presence	Presence

**Table 3:** Modelistic parameters obtained after fitting the data to the best release model (one-site binding model) of the formulations and non-modelistic parameters. Values represent the mean ± SD (*n* = 5).

	One-Site Binding			Statistic
	CSP-O1 ^1^	CSP-O2 ^2^	CSP-O3 ^3^	
Modelistic parameters				
*B_max_* (µg/cm^2^)	628.9 ± 18.2	95.9 ± 2.7	185.6 ± 14.3	*** 1 vs. 2 and 3; *** 2 vs. 3
*K_d_* (h)	3.9 ± 0.4	0.1 ± 0.0	1.2 ± 0.5	*** 1 vs. 2 and 3; ** 2 vs. 3
Non-modelistic parameters				
*AUC* (µg/cm^2^·h)	11,040.0 ± 1087.0	2381.0 ± 248.0	4068.0 ± 403.0	*** 1 vs. 2 and 3; ** 2 vs. 3
*MDT* (h)	6.1 ± 0.5	21.7 ± 2.4	18.7 ± 1.7	*** 1 vs. 2 and 3; * 2 vs. 3
*E* (%)	76.5 ± 7.1	16.5 ± 1.3	28.2 ± 2.6	*** 1 vs. 2 and 3; ** 2 vs. 3

* *p* < 0.05, ** *p* < 0.01, *** *p* < 0.001.

**Table 4:** Estimated permeation and retention parameters of CSP formulations on corneal and scleral membranes in the section where the curves are linear (first hour). Values are reported as the mean ± SD (*n* = 6). *J/sup*: flux normalized by surface; *C_0_*: initial concentration in the donor compartment; *K_p_*: permeability constant; *A*_6_: cumulatively permeated amount of CSP at 6 h; *Q_r_*: amount of CSP retained in the skin.

Corneal Membrane	CSP-O1 ^1^	CSP-O2 ^2^	CSP-O3 ^3^	Statistic
*J/sup* (µg/h/cm^2^)	6.75 ± 0.32	5.46 ± 0.78	43.25 ± 3.49	*** 3 vs 1 and 2
*C*_0_ (µg/mL)	5000	5000	5000	-
*K_p_* (10^−3^ cm/h)	1.35 ± 0.06	1.09 ± 0.16	8.65 ± 0.69	* 1 vs. 3; *** 2 vs. 1 and3
*A*_6_ (µg/cm^2^)	94.99 ± 9.94	27.86 ± 2.84	109.14 ± 12.04	* 1 vs. 3; *** 2 vs. 1 and 3
*Q_r_* (µg/cm^2^)	28.83 ± 2.81	11.47 ± 1.79	7.03 ± 0.75	** 2 vs. 3; *** 1 vs. 2 and 3
Scleral Membrane	CSP-O1 ^1^	CSP-O2 ^2^	CSP-O3 ^3^	Statistic
J/sup (µg/h/cm^2^)	38.40 ± 2.83	13.78 ± 0.81	12.73 ± 0.73	*** 1 vs. 2 and 3
*C*_0_ (µg/mL)	5000	5000	5000	-
*K_p_* (10^−3^ cm/h)	7.68 ± 0.56	2.76 ± 0.16	2.55 ± 0.15	*** 1 vs. 2 and 3
*A*_6_ (µg/cm^2^)	92.32 ± 10.23	66.40 ± 7.32	80.99 ± 8.23	* 2 vs. 3; *** 1 vs. 2
*Q_r_* (µg/cm^2^)	48.79 ± 5.23	30.61 ± 3.47	20.09 ± 2.43	*** 1 vs. 2 and 3; *** 2 vs. 3

* *p* < 0.05, ** *p* < 0.01, *** *p* < 0.001.

**Table 5:** Antifungal activity of the CSP formulations with and without CSP.

Yeast Tested	Origin	Formulations with and without CSP					
		CSP-O1	O1	CSP-O2	O2	CSP-O3	O3
*C. albicans*	ATCC 10231	++	−	++	−	++	+
*C. glabrata*	ATCC 66032	++	−	++	−	++	+
*C. parapsilosis*	ATCC 22019	++	−	++	−	++	+
*C. tropicalis*	ATCC 7349	++	−	++	−	++	+
*C. auris*	DSM 21092	R	−	R	−	R	+

++—formulation totally effective against the strain tested (inhibition zone diameter > 11 mm); +—growth inhibition where samples without the antifungal agent were placed (inhibition zone diameter < 11 mm); −—formulation not effective against the strain tested and no growth inhibition effect was observed; and R—very marked inhibition effect on the yeast growth at 24 h (inhibition zone diameter > 11 mm), but resistant growth appeared within the first inhibition zone at 48 h.

**Table 6:** Irritation Score (IS) of formulations tested with the HET-CAM test.

Formulation	IS Value	Classification
CSP-O1	0.069	N.I.
CSP-O2	0.085	N.I.
CSP-O3	0.113	N.I.

N.I.—no irritating (IS ≤ 0.9); weakly irritating (0.9 < IS ≤ 4.9); moderately irritating (4.9 < IS ≤ 8.9); and irritating (8.9 < IS ≤ 21) [45].

## Data Availability

The data presented in this study are available on request from the corresponding author. The data are not publicly available due to the fact that they are part of a doctoral thesis and it will be available once the thesis is published.
